# Renal Tubule Repair: Is Wnt/β-Catenin a Friend or Foe?

**DOI:** 10.3390/genes9020058

**Published:** 2018-01-24

**Authors:** Leslie S. Gewin

**Affiliations:** 1 The Division of Nephrology, Department of Medicine, Vanderbilt University Medical Center, Nashville, TN 37232, USA; l.gewin@vanderbilt.edu; Tel.: +1-615-343-0767; Fax: +1-615-343-7156; 2 Department of Cell and Developmental Biology, Vanderbilt University, Nashville, TN 37232, USA

**Keywords:** kidney injury, renal fibrosis, epithelial injury

## Abstract

Wnt/β-catenin signaling is extremely important for proper kidney development. This pathway is also upregulated in injured renal tubular epithelia, both in acute kidney injury and chronic kidney disease. The renal tubular epithelium is an important target of kidney injury, and its response (repair versus persistent injury) is critical for determining whether tubulointerstitial fibrosis, the hallmark of chronic kidney disease, develops. This review discusses how Wnt/β-catenin signaling in the injured tubular epithelia promotes either repair or fibrosis after kidney injury. There is data suggesting that epithelial Wnt/β-catenin signaling is beneficial in acute kidney injury and important in tubular progenitors responsible for epithelial repair. The role of Wnt/β-catenin signaling in chronically injured epithelia is less clear. There is convincing data that Wnt/β-catenin signaling in interstitial fibroblasts and pericytes contributes to the extracellular matrix accumulation that defines fibrosis. However, some recent studies question whether Wnt/β-catenin signaling in chronically injured epithelia actually promotes fibrosis or repair.

## 1. Introduction

The human kidney is a highly vascular organ that removes metabolic wastes and excess fluid, maintains acid/base balance, regulates electrolytes, and produces hormones such as renin and erythropoietin that modulate blood pressure and red blood cell production, respectively. Each human kidney contains 500,000–2,000,000 nephrons, the basic functional unit [1]. The nephron is comprised of the glomerulus, which filters the blood, and the tubular epithelium connected to the glomerulus that further modulates water balance and electrolytes. The renal tubule has distinct, highly specialized segments starting with the proximal tubule that connects directly to the glomerulus in the cortex or outer part of the kidney. The proximal tubule leads to the loop of Henle, distal convoluted tubule, and the collecting duct that feeds into the medulla (inner kidney), renal papilla, and eventually the ureter. Due to the high energy requirements, the renal tubules, particularly the proximal tubules, are extremely vulnerable to injury. The tubules have long been recognized as the target of acute kidney injury (AKI) caused by drops in blood pressure, sepsis, toxins, or drugs. In addition, there is growing recognition that the renal tubules can be targeted in chronic renal injuries such as diabetes. The severity and duration of injury as well as how renal tubules respond to injury determines whether the kidney undergoes repair or progresses to tubulointerstitial fibrosis, the accumulation of extracellular matrix proteins that is the hallmark of chronic kidney disease (CKD). Persistent tubular injury and the failure of epithelial repair are increasingly linked to the transition from AKI to CKD and loss of renal function [2,3,4]. Therefore, understanding the pathways that promote renal tubular repair versus fibrosis has important therapeutic implications for halting the progression of CKD, which affects over 13% of the world’s population [5]. 

Wnt/β-catenin signaling in renal injury has attracted much attention both for its potential to promote tubular repair but also as a potential target for fibrosis. There are 19 mammalian Wnt ligands that bind to frizzled and LRP5,6 co-receptors to inhibit the β-catenin destruction complex composed of axin, adenomatous polyposis coli (APC), glycogen synthase kinase-3β (GSK-3β), and casein kinase [6]. In the absence of Wnt ligands, cytosolic β-catenin is targeted for proteosomal degradation by this destruction complex. However, Wnt binding allows for β-catenin stabilization, nuclear translocation, and, through interactions with transcription factors like lymphoid enhancer factor/T cell factor (LEF/TCF), alteration of DNA transcription [6,7]. Wnt/β-catenin signaling regulates many cellular processes such as proliferation, adhesion, differentiation, and survival, which are important to the injured renal epithelia. In addition to its role in signaling, β-catenin serves a structural role as part of the adherens junction complex at the cell membrane. There is also β-catenin-independent or non-canonical Wnt signaling, commonly referred to as the planar cell polarity (PCP) pathway. The non-canonical Wnt signaling pathway is important for oriented cell division and its dysregulation leads to cystic kidneys, but this review focuses specifically on canonical Wnt/β-catenin signaling and renal epithelial injury [8]. 

Several of the Wnt ligands are expressed during renal development, and reporter mice for β-catenin activity show increased activity during nephron development [9,10,11]. In the adult kidney, very little β-catenin activity is present with the exception of collecting duct cells [12], which may have activity due to the hypoxic and hyperosmotic environment of the medulla. However, after renal injury, there is re-expression of Wnt ligands and increased β-catenin activity in both the renal tubules as well as the interstitium. In the unilateral ureteral obstruction (UUO) rodent model, the classic model of tubulointerstitial fibrosis, almost all 19 Wnt ligands are upregulated and β-catenin activity (i.e., nuclear accumulation) is increased after the ureter is ligated [13]. There is also evidence of upregulated Wnt/β-catenin signaling in human renal tubular cells in CKD, but it is unclear whether this signaling pathway is helping promote repair or fibrosis [14]. The conflicting data about whether Wnt/β-catenin signaling is protective in renal injury may be explained by differing target cell types (epithelial versus mesenchymal), different types of acute (acute versus chronic) and different mechanisms of action by systemic inhibitors. This review discusses the role that Wnt/β-catenin signaling plays in the repair and regeneration of the injured renal tubular epithelium. 

## 2. Wnt/β-Catenin Signaling in Kidney Development

Many of the growth factors and gene expression patterns involved in renal development (e.g., Notch, Wnt, hedgehog) are recapitulated after injury as the kidney rebuilds [15]. Thus, an understanding of how Wnt/β-catenin signaling affects renal development may shed insight into its role in renal injury. Development of the permanent kidney (metanephros) in the mouse begins at E10.5, when an outpouching from the mesonephric duct (Wolffian duct) invades the undifferentiated metanephric mesenchyme (MM). The Wolffian duct extension, known as the ureteric bud (UB), undergoes rapid iterative branching to form the ureter, papilla, and collecting ducts. Areas of MM surrounding the invading UB tips condense to form pretubular aggregates, then renal vesicles, and finally epithelialization to form the remaining nephron (glomerulus, proximal tubule down to the collecting duct) [16]. This process of nephrogenesis depends heavily upon interactions between the progenitor cells of the invading UB and MM. 

Wnt ligands play an important role in this epithelial/mesenchymal communication. Expression of Wnt4, Wnt2b, Wnt7b, Wnt6, Wnt9b, and Wnt11 have been detected in the developing kidney, and a LEF/TCF reporter (i.e., β-catenin activity) shows activity in the distal UB tips as well as the adjacent condensing MM [17]. Convincing data illustrate critical roles for Wnt9b and Wnt4 in the developing nephron. Wnt9b is produced by the invading UB and acts in a paracrine fashion to induce a tubulogenic program in MM [18]. Wnt9b initiates MM induction through the effects of downstream Wnt4, expressed in pretubular mesenchyme shortly before aggregation. This Wnt4 expression has been shown to be necessary and sufficient for renal vesicle formation [19,20,21]. Although the final step of epithelialization may require Wnt non-canonical signaling, the interactions of UB-derived Wnt9b and MM-derived Wnt4 to induce nephron formation occur largely through the canonical signaling pathway [20,22]. 

Genetic studies in mice reveal that both β-catenin deletion and over-expression (i.e., stabilization) lead to aberrant renal development. The mechanisms whereby these developmental abnormalities occur provide insights into the effects of Wnt/β-catenin signaling on renal tubular epithelia. Deleting β-catenin specifically in the UB using homeobox B7(Hoxb7)-Cre led to a range of phenotypes from renal agenesis to cystic/dysplastic kidneys with the severity dependent upon the degree of recombination [9,23]. In the cells lacking β-catenin, there was premature expression of aquaporin-3 (AQP3) and zonula occludens -1α (ZO-1α), two markers of differentiated collecting ducts, and early loss of factors associated with precursor cell maintenance (e.g., Sox9) [23]. In a similar study by another group, β-catenin deletion in the UB impaired expression of UB tip genes and reduced branching morphogenesis [9]. Conversely, stabilization of β-catenin in the UB diminished expression of AQP3 [23]. These findings suggest that β-catenin activity in the UB is necessary to maintain a de-differentiated precursor state. 

SIX Homeobox 2(Six2)-Cre was used to selectively delete β-catenin in the MM that led to a smaller nephrogenic zone and fewer nephrons [20]. The remaining nephrons in these β-catenin mutant mice retained β-catenin expression, indicating that β-catenin signaling is required for formation of renal vesicles. Overexpression of β-catenin in the MM caused ectopic tubule induction, but these structures failed to properly epithelialize. This study indicates that β-catenin signaling is necessary for MM to start induction of the tubulogenic program, but downregulation of β-catenin is likely important for epithelial differentiation [20,24]. β-catenin-dependent increases in other growth factors such as glial-derived neurotrophic factor (GDNF) in the MM and TGF-β2 and Dickkopf-related protein 1 (Dkk1) in the UB likely also contribute to the dysplastic phenotypes [25,26]. These genetic studies indicate that β-catenin signaling, both directly and indirectly, is critical for proper differentiation and precursor cell maintenance. 

Wnt/β-catenin signaling is clearly important for renal development, but the amount of signaling as well as spacial and temporal expression are critical. To achieve tight control over Wnt/β-catenin signaling, several modulators of this pathway likely exist. One inhibitor of Wnt in early MM is the transcription factor Six2. The uninduced MM has multipotent progenitor cells, and Six2 was shown to maintain this population in a self-renewing progenitor state, whereas Wnt signaling committed these cells to becoming nephron progenitors [27]. An endogenous inhibitor of Wnt signaling, Dkk1, is expressed in the developing nephrons, and conditional inactivation in the tubules (paired box gene 8 (Pax8)-Cre) caused collecting duct abnormalities characterized by increased proliferation, decreased tubular channel expression, and altered renal function [28]. Supporting the importance of Wnt/β-catenin temporal expression, transient activation using a GSK-3 inhibitor (BIO) in nephron progenitors led to epithelialization [27]. However, if cultures were subconfluent or if BIO treatment was sustained, epithelialization was impaired. Consistent with this, a pulse (but not sustained exposure) of another GSK-3 inhibitor (CHIR99021) was necessary to induce maximal nephron formation in organoids derived from human embryonic stem cells or human induced pluripotent stem cells [29]. Additionally, the duration of CHIR99021 altered the ratio of collecting duct to nephron formation. Therefore, tight regulation of Wnt/β-catenin signaling duration, location, and amount are critical to proper development. Taken together, these data suggest that Wnt/β-catenin signaling plays an important role in renal development mediated primarily through effects on progenitor cell differentiation and nephron commitment. Thus, it is tempting to speculate that Wnt/β-catenin also modulates differentiation and epithelialization in injured renal tubule epithelia. 

## 3. Wnt/β-Catenin Signaling in Epithelial Injury

As mentioned above, Wnt/β-catenin signaling is minimal in the uninjured adult kidney but becomes upregulated in injured tubules during acute and chronic renal injury. Wnt4 and Wnt9b play prominent roles in renal development, but their roles in injured tubular cells may be less important. One study showed increased proximal tubular expression of Wnt4 after ischemia reperfusion (I/R) [30], a classic model of rodent AKI. However, another group marked Wnt4 expression genetically and found that Wnt4 expression was increased in the interstitium but not in renal tubules following injury by either I/R or UUO [31]. Similarly, protein levels of many Wnts, but not Wnt9b, were upregulated in obstructive kidneys [13]. There are several studies supporting upregulated β-catenin activity in injured renal tubules [13,32], but this signaling may be independent of Wnt4 and Wnt9b. The key question to be discussed is whether this epithelial Wnt/β-catenin signaling promotes repair or accelerates tubulointerstitial fibrosis following kidney injury. 

### 3.1. Acute Kidney Injury

Acute kidney injury primarily targets the renal epithelium, particularly the proximal tubules, and several pre-clinical studies indicate that epithelial Wnt/β-catenin signaling plays a protective role early in injury though its role at later time points requires further investigation. Wnt/β-catenin signaling can have potent effects on the epithelial cell cycle which is crucial to repair after AKI. Proximal tubules with Wnt/β-catenin activity also stained for proliferating cell nuclear antigen (PCNA) 24 h after I/R, indicating that these were actively dividing cells [30]. In LLC-PK1 cells, augmenting Wnt/β-catenin activity increased cell cycle progression and was associated with increased activity of cyclin D1, which promotes progression from G1 to S phase [30]. Another group showed that macrophage-derived Wnt7b promoted epithelial repair through effects on proliferation. Macrophage ablation reduced Wnt/β-catenin activity in epithelial cells and reduced regeneration [32]. Furthermore, macrophage-specific Wnt7b deletion also worsened epithelial injury, and Wnt7b was shown to reduce the number of epithelial cells arrested in G2/M, which is associated with a pro-fibrotic phenotype [32]. Thus, Wnt/β-catenin signaling in the acutely injured tubules appears protective by promoting cell cycle progression. 

**Figure 1:**
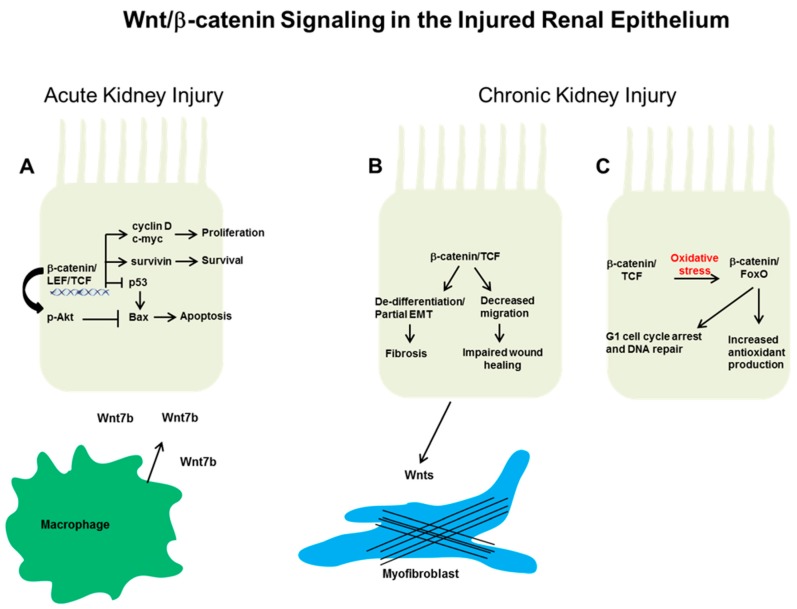
Putative mechanisms whereby epithelial Wnt/β-catenin signaling affects the response to injury. (**A**) After acute kidney injury (Aki) in rodent models, β-catenin signaling can protect against epithelial apoptosis and promote proliferative repair. These actions are thought through β-catenin-dependent transcriptional upregulation of cyclin D, c-Myc, and survivin. In addition, β-catenin suppresses the pro-apoptotic protein Bax (Bcl-2 associated X protein) through blocking p53 and/or by augmenting protein kinase B (Akt) phosphorylation. Macrophage-derived Wnt7b promotes cell cycle progression in injured epithelial cells through paracrine signaling. In chronic kidney injury, the effect of epithelial Wnt/β-catenin signaling on repair or fibrosis is not as clear. (**B**) β-catenin signaling may be detrimental by promoting epithelial de-differentiation or decreased migration, which can lead to increased tubulointerstitial fibrosis and impaired wound healing, respectively. In addition, epithelial-derived Wnts may activate surrounding fibroblasts/pericytes to become myofibroblasts, the main producers of extracellular matrix. Alternatively, (**C**) the high oxidative stress environment of chronic kidney disease (CKD) induces a switch in β-catenin transcriptional binding partners from lymphoid enhancer factor/T cell factor (Lef/Tcf) to forkhead box protein O (FoxO). β-catenin/FoxO have been shown to mediate increased antioxidant production and cell cycle arrest/DNA repair, potentially beneficial responses in other systems but have not been studied in the CKD kidney.

Wnt/β-catenin also mitigates AKI by reducing apoptosis and increasing survival in injured renal tubules. Tubule-specific ablation of β-catenin led to increased injury in mice subjected to two models of AKI: I/R and folic acid nephropathy [33]. These conditional knock-out β-catenin mice had increased tubular apoptosis and expression of Bcl-2 associated X protein (Bax), a pro-apoptotic Bcl-2 family protein that mediates mitochondrial injury [33]. In addition, β-catenin conditional knockout mice had increased p53 as well as decreased phospho- protein kinase B (Akt) and survivin, indicating many different pathways whereby β-catenin may protect against tubular apoptosis as shown in Figure 1A. Another group investigated β-catenin’s anti-apoptotic effects on proximal tubule cells using constitutively active and dominant negative constructs in vitro. Similar to the in vivo study, β-catenin reduced Bax activation, translocation to the mitochondria, mitochondrial membrane injury and apoptosis in a PI-3K/Akt-dependent pathway [34]. One caveat is that β-catenin deletion affects its structural role as part of the adherens junction complex in addition to its signaling role. Although β-catenin deletion did not cause any detectable adherens junctions abnormalities (γ-catenin, or plakoglobin, undergoes compensatory upregulation) in mice [33], it is possible that subtle structural abnormalities contribute to the phenotype. 

Several groups have shown that suppressing GSK-3β, the protein that targets β-catenin for proteosomal degradation, also mitigates AKI. Genetic inhibition of GSK-3β in murine proximal tubules reduced apoptosis and mortality following HgCl_2_-induced AKI [35]. Furthermore, treatment with TDZD-8, a GSK-3 inhibitor, two days after AKI accelerated renal recovery and proliferation [35]. TDZD-8 also reduced renal injury in rats injured by I/R by inhibiting GSK-3β induced Bax activation and apoptosis [36]. Another group used lithium to block GSK-3β either three days after cisplatin administration or eight hours after I/R [37]. This delayed lithium administration improved renal repair and functional recovery after AKI and was associated with increased nuclear cyclin D1, c-Myc, and Hif-1α [37]. It is tempting to assume that these GSK-3 inhibitors may be beneficial, at least in part, through β-catenin activity as similar targets (Bax, cyclin D1) are affected. However, most of these studies did not analyze the extent to which these GSK-3 inhibitors were affecting β-catenin activity (e.g., nuclear expression rather than total expression), and this is important as GSK-3 inhibitors have β-catenin-independent effects, too. Therefore, the role of β-catenin in mediating the protective effects of GSK-3 inhibition in AKI requires further investigation. Even with the limitations mentioned, the data overall support a compelling protective role of Wnt/β-catenin in early epithelial injury and repair (Table 1). 

### 3.2. Wnt/β-Catenin Signaling in Tubular Progenitors after Injury

During AKI, tubular epithelia are susceptible to apoptosis/necrosis, and there is much interest in understanding how tubular repair and replacement of these lost cells occurs. Many groups are successfully differentiating human embryonic stem cells to form renal tubules and other structures, which has exciting promise in the area of tissue regeneration [41,42]. However, in vivo, there is accumulating evidence that tubular epithelial cells lost in injury are replaced by other proliferating tubular cells. Lineage tracing studies showed that fluorescently labelled proximal tubules were the source of replacement tubule cells after AKI [43,44]. Another group has shown that, both in homeostasis and following injury, new tubules are produced by clonal expansion of segment-restricted tubules [45]. This study used the “rainbow” mice in which cells express one of four colors determined randomly by recombination with all progeny cells maintaining the parental color expression, thus defining clonal expansion. After murine I/R and glycerol-induced AKI, several clonal expansions occurred, but they did not cross tubule segments (e.g., proximal tubule, distal convoluted tubule) [45]. This implies that a subset of cells within a particular tubular segment expands to replace injured cells. One controversy in the field is whether these proliferating renal tubules come from a fixed subset of tubule cells with unipotent progenitor capacity or if all tubular cells, upon de-differentiation, have the capacity for this clonal expansion [46]. However, there is agreement that segment-restricted, unipotent precursors repair the injured tubular epithelia. 

Wnt/β-catenin signaling likely plays an important role in these proliferating tubular epithelial cells. Wnt-responding cells were lineage traced by using a tamoxifen-inducible Cre under the axis inhibition protein 2 (Axin2) promoter crossed with the mT/mG “tomato” reporter mouse to mark Wnt-responsive (i.e., active AXIN2 promoter) cells with GFP [45]. After AKI, many GFP positive clones expanded in a segment-restricted manner. Another group also showed upregulation of Wnt/β-catenin signaling using the TCF-lacZ reporter mice, and many of these β-galactosidase positive cells also expressed the renal progenitor marker CD24 [47]. Furthermore, this group showed that CD24 positive renal progenitor cells could engraft in injured tubules, but blocking canonical Wnt signaling impaired this process. Thus, just as Wnt/β-catenin signaling is important in the developing nephron, this pathway is also active in tubular progenitors that are integral to epithelial repair. Defining which Wnt/β-catenin-dependent effects (e.g., proliferation, apoptosis, differentiation) contribute to repair by tubular progenitors requires further investigation. 

## 4. Wnt/β-Catenin Epithelial Signaling in Chronic Kidney Disease

### 4.1. Systemic Inhibitors of Wnt/β-Catenin 

Wnt/β-catenin signaling is increased in injured epithelia from both murine CKD models and human biopsies. Unlike AKI, the role of this signaling in repair versus fibrosis progression is less clear. Many studies examining Wnt/β-catenin in rodent models of CKD have done so using systemic inhibitors which have shown improvement in tubulointerstitial fibrosis [48,49,50,51,52]. Although these findings imply that Wnt/β-catenin signaling promotes fibrosis, they do not shed light on how this pathway specifically modulates epithelial responses to injury. Wnt4 is upregulated in myofibroblasts in CKD models and drives differentiation of pericytes into myofibroblasts [31]. Genetic studies either overexpressing or inhibiting Wnt secretion by renal tubular epithelia show that the primary effect is on paracrine β-catenin signaling in neighboring mesenchymal cells [38,51,53]. Thus, it is very likely that systemic inhibitors of Wnt/β-catenin signaling exert their protective effects by blocking signaling in the myofibroblasts rather than tubular epithelia. 

Another complicating factor regarding systemic Wnt/β-catenin inhibitors is that certain widely used inhibitors such as ICG-001 actually modulate rather than block signaling. ICG-001 selectively inhibits β-catenin’s interactions with CBP, and recent work with Tregs also demonstrates reduced β-catenin binding to TCF [54,55,56]. Classically, β-catenin binds with transcription factors LEF/TCF to mediate its effects (e.g., proliferation, de-differentiation). However, several groups have shown that, under oxidative stress, β-catenin switches binding partners from LEF/TCF to FOXO [57,58,59,60]. FOXO transcription factors modulate stress responses such as DNA repair, G1 cell cycle arrest, and antioxidant production, which may be protective in CKD [61,62]. ICG-001 has been shown to reduce β-catenin/TCF-dependent cellular effects and augment β-catenin/FOXO [54]. It is possible that β-catenin/LEF/TCF interactions are deleterious in renal tubular injury, but β-catenin/FOXO transcriptional responses may be beneficial. If so, the beneficial effects of ICG-001 in renal injury, a condition of oxidative stress, may be due to augmentation of β-catenin/ FOXO rather than inhibition of all β-catenin-dependent signaling. The role of β-catenin/ FOXO transcriptional responses in renal epithelial injury has not been well-studied and is the focus of ongoing research by our group. 

### 4.2. Epithelial Wnt/β-Catenin and Chronic Kidney Disease

Given the divergent effects of Wnt/β-catenin on epithelial cells versus fibroblasts, studies examining this signaling pathway specifically in injured renal epithelia are particularly informative. Tubule-specific deletion of β-catenin (using kinesin spindle protein (Ksp)-Cre) did not have much effect on renal fibrosis after the UUO model of CKD [39]. These conditional knockout mice did have enhanced survival of interstitial fibroblasts due to decreased MMP-7-dependent Fas ligand expression. As mentioned earlier, the concern that β-catenin is part of the adherens junction complex as well as a transcriptional mediator of epithelial responses applies to this study. In addition, the UUO model is an efficient producer of fibrosis but may not be the ideal model in which to examine epithelial injury since there is no opportunity for repair (progressive destruction of renal parenchyma) or measurement of functional parameters (uninjured contralateral kidney compensates). Nevertheless, this important study suggests that epithelial β-catenin activity is not a significant driver of tubulointerstitial fibrosis in the UUO model. 

Several β-catenin-mediated epithelial effects that have been demonstrated in vitro might promote tubulointerstitial fibrosis (Figure 1B). Wnt/β-catenin signaling promotes loss of epithelial markers like E-cadherin and gain of mesenchymal protein expression (e.g., vimentin), known as epithelial to mesenchymal transition (EMT), through β-catenin/LEF-1 activity in vitro [63,64]. Although complete EMT arguably does not occur in CKD, a process of de-differentiation or partial EMT is present in injured epithelia and associated with progression of tubulointerstitial fibrosis [65,66,67]. Another group has shown that β-catenin signaling impaired renal tubular migration and wound healing under hypoxia in vitro [68]. It is possible that Wnt/β-catenin signaling is important for repair early in injury, but sustained signaling may promote fibrosis over repair. This would be similar to β-catenin’s role in development, where it is necessary for nephrogenesis initiation, but prolonged β-catenin activity impairs epithelialization. However, it is also possible that many of the deleterious in vitro effects of β-catenin are mediated through its interactions with LEF/TCF, which may be reduced in the highly oxidative environment of CKD in favor of binding with FoxO (Figure 1C). 

There is some additional evidence that epithelial Wnt/β-catenin signaling may be protective in CKD. Wnt6 expression was dysregulated in the tubulointerstitium of patients with diabetic nephropathy and tubules of mice after UUO [69]. In vitro, Wnt6 activated the canonical signaling pathway, enhanced tubulogenesis of MDCK cells, and blunted TGF-β-induced de-differentiation and NF-κB activation [69]. These in vitro data suggest that epithelial Wnt6 may be protective but further genetic manipulation in CKD models is necessary to confirm. Our group recently showed that conditional stabilization (i.e., activation) of β-catenin in the proximal tubule reduced epithelial injury and renal fibrosis [40]. However, there are two important limitations: these studies were performed on mice that also lacked the TGF-β receptor in the proximal tubule. As TGF-β and Wnt/β-catenin pathways intersect at multiple levels, future studies should investigate the role of β-catenin stabilization in mice with TGF-β signaling intact. Second, most CKD models involve a component of acute injury, so inducible β-catenin stabilization after the acute phase of injury would better assess epithelial β-catenin’s effects on CKD progression. Thus, more research is necessary to define how Wnt/β-catenin signaling in renal epithelia alters the response to chronic injury. 

## 5. Therapeutic Implications and Future Directions

In summary, the Wnt/β-catenin signaling pathway is critical for proper nephron development and is re-activated in the injured renal tubular epithelium and, specifically, the proliferating tubular progenitors that repopulate the tubule after injury. Several studies suggest that epithelial Wnt/β-catenin is protective in the context of AKI, potentially through anti-apoptotic or pro-proliferative effects. Whether epithelial Wnt/β-catenin is friend or foe in CKD requires further study, and the answer likely depends upon temporal factors and/or the amount of signaling. There may be an important dose-dependent effect whereby a modest increase in epithelial Wnt/β-catenin signaling is beneficial, but augmenting this pathway too much promotes a fibrotic response. It is also important to understand that various inhibitors (e.g., GSK-3β inhibitors and ICG-001) modulate Wnt/β-catenin signaling rather than cleanly augment or suppress this pathway. To clarify the role of Wnt/β-catenin signaling in chronically injured epithelia, future studies should alter this signaling pathway in a cell-specific way after the acute phase of injury so that its role in acute versus chronic injury can be discerned. Additionally, if Wnt/β-catenin signaling is enhanced, it should be done so in a way that just affects epithelial signaling (as activity in fibroblasts is clearly detrimental), and graded responses should be measured to consider potential dose-dependent responses.

Other signaling pathways such as TGF-β and the renin-angiotensin system, also upregulated in chronic injury, have been shown to augment Wnt/β-catenin signaling [40,49,70,71,72]. Additional studies are necessary to determine if these important upstream regulators can differentially affect β-catenin transcriptional responses. In addition, future research should clarify whether CKD-induced oxidative stress alters β-catenin’s transcriptional responses from LEF/TCF-dependent to FoxO-dependent. If so, defining the targets of β-catenin/FoxO interactions may yield novel therapeutic approaches. Furthermore, Wnt antagonists are being investigated as potential therapies for CKD given the profibrotic effects of β-catenin signaling in interstitial myofibroblasts. Understanding how blocking this pathway affects the injured epithelia will be important for designing safe antifibrotic therapies. 

## Figures and Tables

**Table 1:** Genetic manipulation of Wnt/β-catenin signaling and its effects (direct and indirect) on renal tubular epithelia. Studies that have genetically altered the Wnt/β-catenin signaling pathway either directly in renal tubules or indirectly (with effects on renal tubules) are listed with the injury models used, response of the conditional knockout or transgenic mouse, putative mechanism, and the appropriate reference. Of note, while the response noted is in vivo, the mechanism may be based on in vitro experiments. Studies manipulating Wnt/β-catenin signaling in the glomerular epithelial cells (podocyte, parietal epithelium) were beyond the scope of this review. Several additional studies have looked at Wnt/β-catenin in the injured kidney using systemic inhibitors, but these were not included, as they do not.

Manipulation of Wnt/β-Catenin Pathway	Injury Model	Response	Mechanism	Ref.
Deletion of Wnt7 in macrophages	I/R	Increased renal tubular apoptosis	Cell cycle progression and basement membrane repair	[32]
Tubule specific ablation of β-catenin	I/R and folic acid	Greater mortality and tubular apoptosis, lower renal function	Increased pro-apototic Bax and p53, decreased pAkt and survivin	[33]
GSK-3β inhibition in proximal tubule	HgCl_2_	Reduced tubular apoptosis and mortality, improved function	Increased cell proliferation and cyclin D1/c-Myc	[35]
Wnt1 overexpression by proximal tubule	None	Increased interstitial fibrosis	Increased paracrine myofibroblast signaling, no epithelial injury	[38]
Tubule specific ablation of β-catenin	UUO	No effect on fibrosis, reduced epithelial de-differentiation, increased fibroblast survival	Fibroblast survival due to reduced MMP-7-dependent FasL induction	[39]
Proximal tubule specific β-catenin stabilization (cells also lack TGF-β receptor)	Aristolochic acid	Reduced tubulointerstitial fibrosis, improved renal function, and reduced tubular injury	Reduced susceptibility to apoptosis and decreased G2/M arrest	[40]
Mutation of Frizzled4 receptor, primarily expressed in epithelia	I/R	Persistent epithelial injury	Increased apoptosis	[32]

I/R: ischemia/reperfusion; GSK: glycogen sythase kinase; UUO: unilateral ureteral obstruction; MMP-7: matrix metalloproteinase-7; TGF-β: transforming growth factor-β.
